# Phantom-Enhanced High Mechanical Accuracy for Frame-Based Deep Brain Stimulation

**DOI:** 10.7759/cureus.66025

**Published:** 2024-08-02

**Authors:** Mark Sedrak, Patrick Pezeshkian, James Latoff, Siddharth Srivastava, Ross W Anderson

**Affiliations:** 1 Neurosurgery, Kaiser Permanente, Redwood City, USA; 2 Neurosurgery, Stanford University, Palo Alto, USA; 3 Neurosurgery, Stanford University Hospital, Palo Alto, USA; 4 Neuroscience and Neurosurgery, Kaiser Permanente, Redwood City, USA

**Keywords:** frame-based stereotaxis, crw system, targeting accuracy, stereotactic neurosurgery, deep brain stimulation

## Abstract

Deep brain stimulation (DBS) is a neurosurgical procedure that depends on high-accuracy targeting of structures to implant electrodes within the brain. The positioning of these electrodes in the brain determines the long-term efficacy of treating diseases such as Parkinson's disease, essential tremor, or dystonia. Misplaced electrodes in DBS can lead to poor efficacy and stimulation-induced side effects. Widespread targeting errors and variability have been published throughout the literature. As such, improvement in targeting accuracy is needed to enhance the quality of the procedures.

A stereotactic phantom was utilized to test and adjust targeting before the surgical placement in the brain for 91 sequential electrodes. The tip of the microelectrode, the first rigid point in time, was measured and compared to the planned target. The technique utilized a to-target cannula with an XY stage that allowed x-axis and y-axis adjustments and correction for inaccuracies relative to the phantom. A calculation was developed to convert anatomical angles (sagittal and coronal) provided by commercial planning stations to spherical angles to calculate points along a trajectory. Error calculations included each dimensional axis, Euclidean error, and radial error.

Bends in the cannula and microelectrode were observed and corrected by referencing the phantom. All 91 first-pass tracks traversed the intended target, and three electrodes required a second mapping track beyond the first penetration due to neurophysiological and intraoperative testing. The results showed overall ultra-high (0-0.5 mm) to high (>0.5-1 mm) accuracy, an average Euclidean error of 0.66±0.30 mm, and a radial error of 0.45±0.28 mm with dimensional errors of less than 0.5 mm per axis.

The utilization of a stereotactic phantom is an important tool to enhance the stereotactic targeting before insertion into the brain. This phantom technique yielded ultra-high to high accuracy in error calculations. Future methods and studies should focus on error minimization to enhance these DBS mechanical accuracy and correlations with clinical outcomes.

## Introduction

Deep brain stimulation (DBS) is a sophisticated neurosurgical procedure that involves the insertion of electrodes into specific targets within the brain. This technique is employed to modulate neural activity and has shown significant efficacy in treating various neurological disorders such as Parkinson's disease, essential tremor, and dystonia. The success of DBS largely hinges on the accuracy of electrode placement, which directly influences therapeutic outcomes and minimizes adverse effects [1-3].

Several techniques have been developed for DBS surgery over the last several decades. These include using a stereotactic frame, which provides a stable reference for targeting; robotic assistance, which offers high precision and repeatability; frameless techniques, which allows for neck mobility and patient comfort; and intraoperative magnetic resonance imaging (iMRI), which allows for real-time visualization of the brain during surgery. Each of these methods has its advantages and limitations. Despite advancements in technology, widespread targeting errors in DBS have been reported in the literature [4-6]. These errors and the variability in electrode placement have been documented across numerous studies. Many publications focus on the discrepancy between the intended target and the final position of the DBS electrodes. However, this endpoint analysis does not provide comprehensive insights into the underlying causes of these inaccuracies. Factors contributing to endpoint accuracy include the mechanical accuracy of the stereotactic system, brain shift during surgery, electrode curvature or migration, image fusion errors, and pixel size in imaging modalities [7].

In this study, we investigated the mechanical accuracy of a frame-based stereotactic system. A stereotactic phantom was referenced for the targeting, and a method was developed around this tool that allowed for corrections before insertion into the brain. By observing the position of the rigid microelectrode tip in the brain relative to the intended target, we aimed to minimize the confounding effects of endpoint analysis and isolate the mechanical accuracy of the system. Finally, we sought to calculate the errors and categorize the accuracy.

## Technical report

Phantom technique

**Figure 1:**
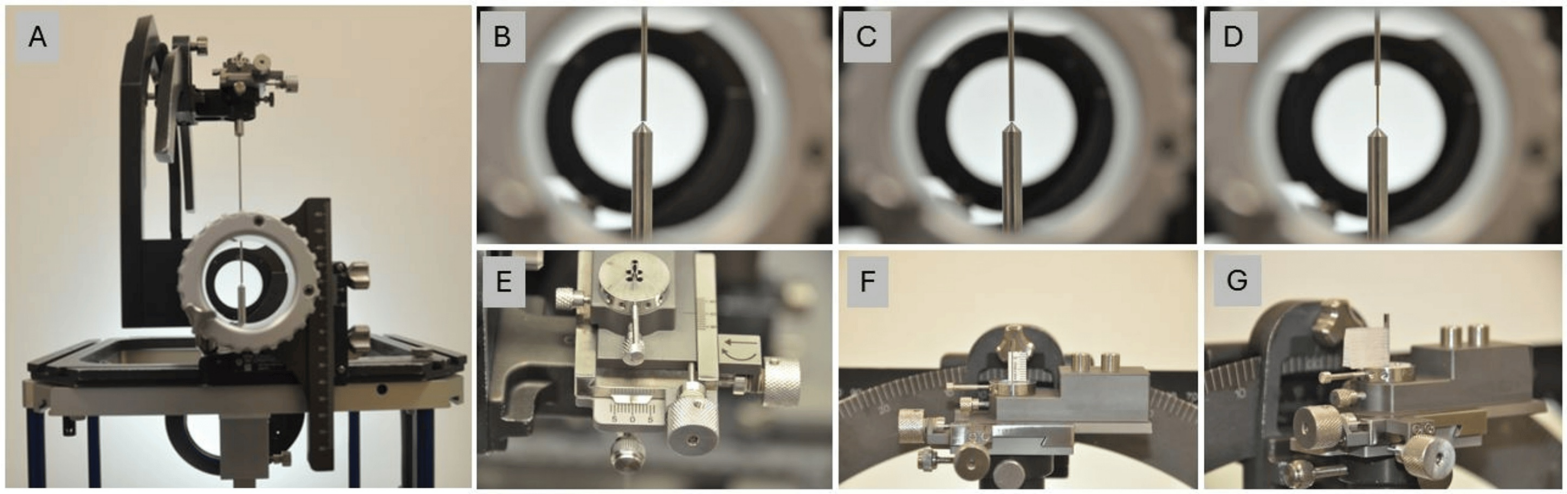
Phantom technique with the demonstration of cannula and microelectrode calibration adjustments. First, the CRW phantom and the arc are dialed to the same coordinates and affixed with the XY stage and to-depth cannula (A). Next, the tip locations are analyzed, and the XY stage is used to adjust the cannula tip to the phantom tip (B and C). The to-depth cannula is withdrawn by 12 mm, and the microelectrode can be measured (D). Views of the XY stage adjustment (E), measured in a 12 mm ruler (F), and a Steri-Strip (G) to hold the cannula at the designated depth. After microelectrode recordings are completed, the to-depth cannula can be placed on the target, followed by the insertion of the deep brain stimulation electrode. CRW: Cosman-Roberts-Wells

In this cohort, the Cosman-Roberts-Wells (CRW) (Radionics CRW Stereotactic System, Integra LifeSciences Corporation, Plainsboro, NJ) frame was utilized during DBS surgeries. A registration computed tomography (CT) scan was obtained (64-slice Discovery HD750 CT Scanner, General Electric, Boston, MA) for localization using a head fixation apparatus previously published [8]. During surgery, a CRW phantom and precision arc were set and affixed. Next, the Alpha Omega (AO) (Alpha Omega Engineering, Nof HaGalil, Israel) XY stage was placed on the arc, and a to-depth cannula was checked against the phantom (Figure 1). XY stage adjustments ($\Delta$X and $\Delta$Y) were made under the assumption that the phantom had fewer mechanical errors than the variables associated with the arc. In many instances, the AO to-depth cannula was rejected due to obvious precession/bends when rotating by greater than approximately 0.5 mm. The cannula was only used when the XY stage adjustments were approximately <0.5 mm in the x-axis and/or y-axis. The cannula was marked with a sterile marking pen indicating the direction of insertion relative to the XY stage. Next, the cannula was withdrawn to 12 mm above the target, and a Steri-Strip (Steri-Strip™ Reinforced Adhesive Skin Closures, 3M™, Saint Paul, MN) was used to hold this position. The microelectrode was then placed and rejected for high bend; the direction of insertion was marked with a marking pen on the apparatus. During the surgery, an image is taken using a cone-beam CT (O-arm, Medtronic, Minneapolis, MN) at the isocenter while in the brain. After microelectrode recordings are completed, the to-depth cannula can be placed on the target, followed by the insertion of the deep brain stimulation electrode.

Stereotactic phantom calibration

**Figure 2:**
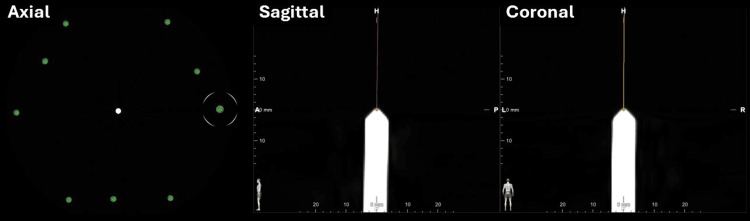
Phantom calibration in computed tomography using localizer. Demonstration computed tomography (CT) images from left to right are axial, sagittal, and coronal, showing the tip of the phantom calibration tool set to coordinates (0,0,0), calibrated using the mounted stereotactic localizer and an artificial trajectory using a commercial software set to the tip. This analysis was performed using multiple phantoms showing an anteroposterior (AP) average error of 0.175/0.075 mm and lateral (LAT) average error of 0.025/0.2 mm. Images are from Brainlab Elements (Brainlab, Inc, Feldkirchen, Germany).

As the CRW phantom was used as a reference for the stereotactic arc and instrument correction using the XY stage, it should be noted that it can also be tested and even calibrated. This is done by setting the phantom to a coordinate such as (0,0,0) and then affixing the localizer frame (Brown-Roberts-Wells Localizer Frame {BRWLF}) and obtaining a CT (Figure 2). Using commercial software, standard localization can be accomplished, and the position of the phantom pin is calculated by placing a trajectory target at the tip. While pixel size and localization methods can affect accuracy, this is an excellent means for quality checks on the equipment being used. The phantom can be set to several coordinates, and this procedure can be repeated throughout the stereotactic space. This analysis was performed using multiple phantoms showing an average error in anteroposterior (AP) of 0.175/0.075 mm, lateral (LAT) of 0.025/0.2 mm, and vertical (VERT) of 0.2 mm, within 0.5 mm of the manufacturer's specifications.

Dimensional, Euclidean, and radial error calculations

**Figure 3:**
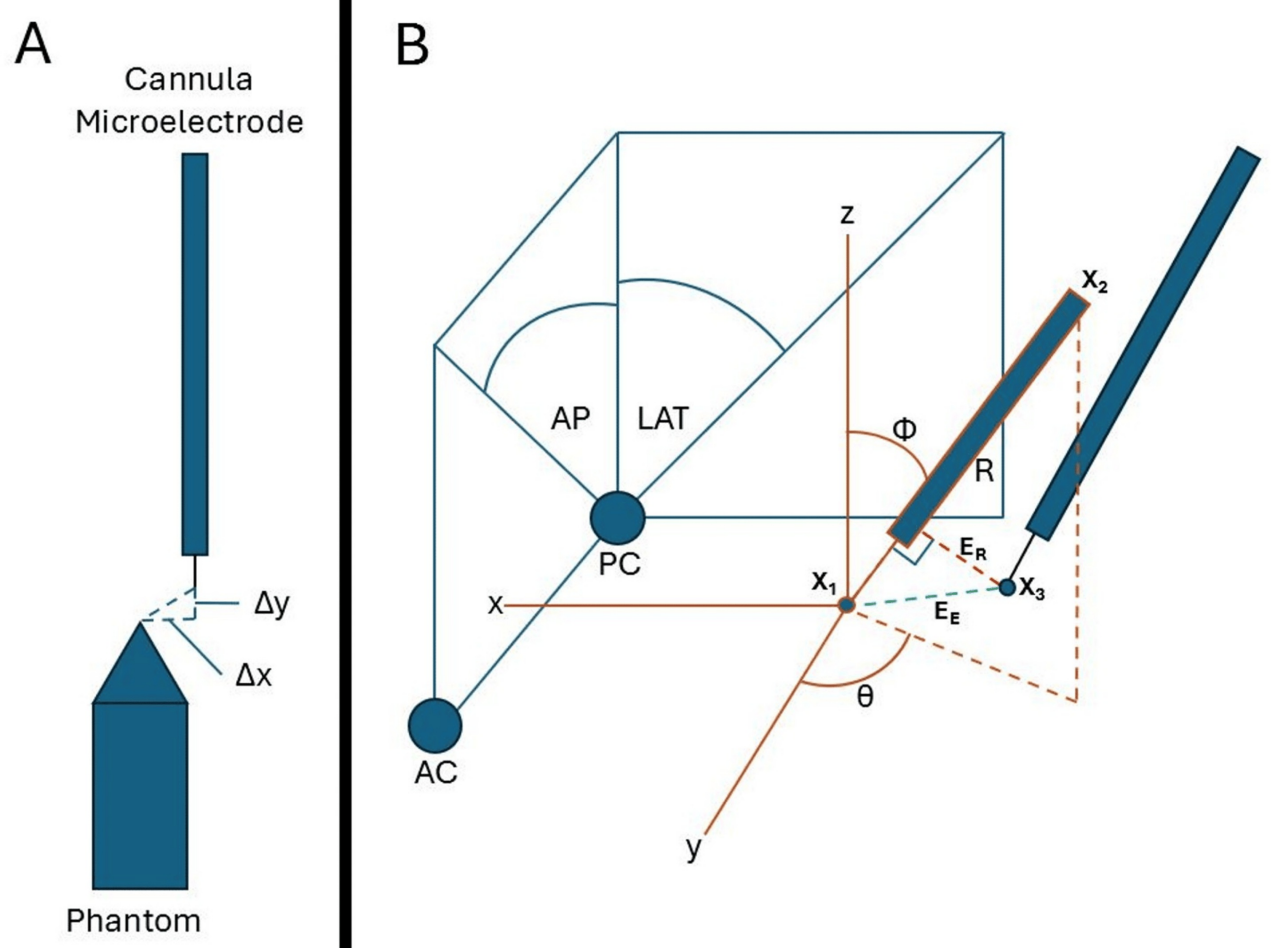
Corrections to microbends in deep brain stimulation (DBS) cannula and microelectrode can be accomplished by the use of a phantom to correct for the misalignment of the stereotactic arc system (ΔX and ΔY) (A). The utilization of anatomical anteroposterior (AP) and lateral (LAT) angles relative to the anterior commissure (AC) and posterior commissure (PC) space provided in commercial software can be converted to spherical coordinates φ and θ (B). For any given surgery, targeting error can be calculated by using the planned target X₁ and entry X₂ relative to the intraoperative location X₃. X₂ can be any point along a trajectory, such as at the level of the skull. With these three-dimensional data, dimensional errors in each axis can be calculated in addition to Euclidean E_E and radial E_R errors. Finally, R is the distance from X₁ to X₂ along a trajectory.

Using the three-dimensional (3D) data, we can calculate errors in each dimension, as well as Euclidean $E_E$ and radial $E_R$ errors relative to the intended initial trajectory and target (equations 1 and 2). $X_1 = x_1, y_1, z_1$ is the initially planned target point, $X_2 = x_2, y_2, z_2$ is an initially planned entry point along the trajectory (e.g., at the dura or skull), and $X_3 = x_3, y_3, z_3$ is the new point measured outside the planned trajectory, such as the microelectrode tip in the brain (Figure 3). Euclidean error $E_E$ is calculated using the Pythagorean theorem in 3D. The radial error $E_R$ is calculated mathematically as the formula for the shortest distance from a point $X_3$ to a line defined by two points $X_1$ and $X_2$. This formula is derived from the cross-product of vectors and the Pythagorean theorem. It calculates the perpendicular distance from the point to the line:

$$E_E = \sqrt{(x_2 - x_1)^2 + (y_2 - y_1)^2 + (z_2 - z_1)^2} \tag{1}$$

$E_R = \frac{\sqrt{((y_2 - y_1)*(z_3 - z_1) - (z_2 - z_1)*(y_3 - y_1))^2 + ((z_2 - z_1)*(x_3 - x_1) - (x_2 - x_1)*(z_3 - z_1))^2 + ((x_2 - x_1)*(y_3 - y_1) - (y_2 - y_1)*(x_3 - x_1))^2}}{\sqrt{(x_2 - x_1)^2 + (y_2 - y_1)^2 + (z_2 - z_1)^2}} \tag{2}$

 

 

 

 

 

A method for the conversion of anatomical angles to spherical coordinate angles

The calculation of points along a trajectory can facilitate other computations and can be determined using a target point and anatomical angles. In commercial stereotactic planning software, such as Brainlab Elements (Brainlab, Inc., Feldkirchen, Germany), the entry point for a planned trajectory is not given but can be determined by picking a point along a trajectory. The software does provide anatomical angles relative to the anterior commissure (AC) and posterior commissure (PC) named sagittal angle (SA) and coronal angle (CA) along with the target point in anatomical coordinates. Therefore, we will describe a method for converting anatomical angles (SA and CA) to spherical coordinate angles azimuth $\Theta$ and zenith $\Phi$, and then using these angles, we can compute a point, such as the entry, along a trajectory. Figure 3 includes these angles, as well as a coordinate system with a planned target point labeled $X_1$, an entry point labeled $X_2$, and a new trajectory point labeled $X_3$. It also depicts angles $\Theta$ and $\Phi$ relative to the coordinate axes.

To convert SA and CA to azimuth $\Theta$ and zenith $\Phi$, we use the following procedures and geometric relationships. First, the equation for the distance from the origin to the point along the trajectory is $R$ given by equation 3, based on the Pythagorean theorem:

$$R^2 = X^2 + Y^2 + Z^2 \tag{3}$$

Next, from Figure 3, the following trigonometric relationships can be determined relative to the angles and axes, giving equations 4-7:

$$tan(CA) = X/Z \tag{4}$$

$$tan(SA) = Y/Z \tag{5}$$

$$\cos(\Phi) = \frac{Z}{R} \tag{6}$$

$$tan(\Theta) = X/Y \tag{7}$$

Therefore, for converting anatomical sagittal angle (SA) and coronal angle (CA) to spherical angles azimuth $\Theta$ and zenith $\Phi$, equations 8 and 9 can be derived from equations 3-7. It should be noted that some CA and SA values may not be converted to $\Theta$ and $\Phi$ because of the use of standard tangent and resultant calculation errors:

$\Theta = \arctan\left(\frac{\tan(CA)}{\tan(SA)}\right)\tag{8}$

 

$$\Phi = ACOS\left(\frac{1}{\sqrt{tan(SA)^2 + tan(CA)^2 + 1}}\right) \tag{9}$$

Once the spherical coordinate angles are calculated, they can be more easily used to get a point along a trajectory from a planned target $X_1$ using equations 10-12:

 

$$x_3 = R*cos(\Theta)*sin(\Phi)+x_1 \tag{10}$$

 

$$y_3 = R*sin(\Theta)*sin(\Phi)+y_1 \tag{11}$$

 

$$z_3 = R*cos(\Phi)+z_1 \tag{12}$$

Results

**Figure 4:**
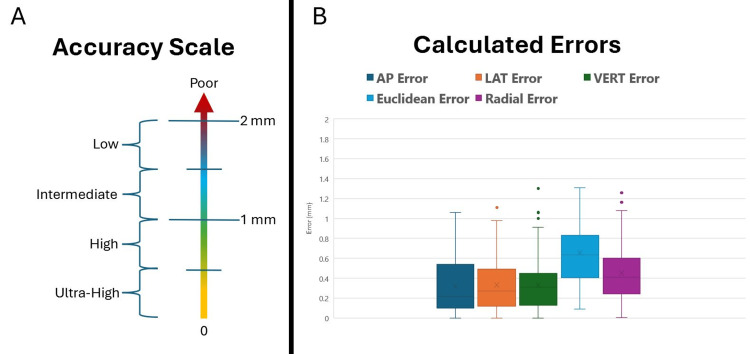
An accuracy scale is categorized by their scalar magnitude in millimeters where 0-0.5 mm is ultra-high, > 0.5-1 mm is high, > 1-1.5 mm is intermediate, > 1.5-2 mm is low, and > 2 is poor (A). Calculated errors in the targeting of the microelectrode tip relative to the intended target that are calculated as dimensional, Euclidean, and radial errors are displayed as a box plot (B). Most errors are ultra-high to high, with a few intermediate-level errors. Box plot computed with Microsoft Excel (Microsoft Corp., Redmond, WA). AP, anteroposterior; LAT, lateral; VERT, vertical

All 91 first-pass tracks traversed the intended target of object segmentation using Brainlab Elements; three electrodes required a track beyond first penetration due to neurophysiological and intraoperative testing. The targeting errors were computed in terms of dimensional errors, which are the scalar differences in anteroposterior (AP), lateral (LAT), and vertical (VERT), as well as Euclidean error $E_E$ (equation 1) and radial error $E_R$ (equation 2). The planned target was used as $X_1$, the tip of the microelectrode was used as $X_3$, and $X_2$ was calculated using the anatomical angles as described in the prior section using an $R$ of 70 mm with $X_1$. The results showed an average $E_E$ of 0.66±0.30 mm and $E_R$ of 0.45±0.28 mm (Figure 4). Using the suggested accuracy scale depicted in Figure 4, most errors are ultra-high to high, with a few intermediate-level errors. It can be seen that $E_E$ is the most conservative measure of error resulting in the highest value.

## Discussion

The literature demonstrates a wide variation of targeting accuracy. However, a fundamental concept is ensuring the mechanical accuracy of the system in use. Therefore, the first position in the brain should closely match the intended target. During DBS surgery, the indented target can change by varying depth or with other radial adjustments. After a DBS electrode is implanted, the electrode position can appear to move in the brain due to decannulation, brain shift, image fusion, and electrode curvature, as well as limitations of resolution due to pixel size. These issues are a focus of a later study and beyond the scope of evaluating the mechanical accuracy of the system. In addition, we have previously published on stereotactic intraoperative localization (StIL), as well as utilizing different coordinate systems during DBS surgery [9,10]. These intraoperative tracking methods provide a secondary method of calculation that can be complementary to 3D imaging/fusion techniques. Other factors that may improve targeting accuracy include head stabilization during imaging, localizer techniques (geometry and multi-slice enhancements), and imaging reconstructions. Further, initial targeting accuracy is felt to be important because small adjustments of <2 mm in the brain can be difficult to accomplish due to falling into the same track, making the first puncture the most critical. In this review, we have focused on anatomical coordinates and discussed a method to convert AP and LAT angles into spherical coordinate angles with dimensional errors per axis, a point-to-line calculation for radial error, and Euclidean error.

This study points to the ability to correct instruments, such as the stereotactic frame and microbends in the cannula and microelectrode during DBS surgeries. These adjustments could also be the result of slight inaccuracies of the arc, XY stage, or interfaces between the materials. Therefore, an external check before insertion in the brain is needed to prevent inaccurate electrode placement. The importance of the phantom is highlighted in this review, and it was also noted that the phantom accuracy could also be measured using the same stereotactic localization method used for the patient in Figure 2, which is a novel engineering achievement. There may be other methods that can be used to correct for these factors such as the use of a rigid ruler and the extrapolation of a trajectory, but a key issue is correcting errors prior to use. Intraoperative MRI provides a unique opportunity to direct DBS targeting by making adjustments to the trajectory during the operation without multiple brain penetrations; however, this comes with the expense of performing the surgery without intraoperative testing and the significant expense of the infrastructure. Notably, many other stereotactic systems do not have a phantom system.

It is known that misplaced DBS electrodes outside of an intended target can result in poor efficacy and stimulation-induced side effects. However, improved programming techniques such as image-guided programming can improve clinical outcomes in deep brain stimulation as long as the electrodes are close to the intended target [11]. These small electrical field adjustments demonstrated by image-guided programming support the notion that high accuracy is also likely to improve outcomes. Therefore, optimizing the surgical accuracy and the programming techniques is likely to have an additive effect. Given the relatively small targets in the brain, such as the subthalamic nucleus, it is difficult to argue against the need to impose the utmost care to achieve the highest possible accuracy. As often quoted by the late professor Lars Leksell, "The tools used by the surgeon must be adapted to the task, and where the human brain is concerned, they cannot be too refined" [12]. As such, future methods and studies should continue to focus on error minimization to enhance quality and outcomes.

## Conclusions

Using a phantom technique with a frame-based stereotactic system, there is a high mechanical accuracy for 91 electrodes. A phantom for stereotactic surgery may be an important tool to test and correct instruments during surgery, and it can undergo calibration and quality assessment. This study focused on evaluating the mechanical accuracy of a frame-based stereotactic system by observing the rigid position of the microelectrode tip relative to the intended target and did not evaluate other factors that lead to the final endpoint position of a DBS electrode relative to the intended target, which is a more commonly cited evaluation. There is likely a correlation between the rigid position of the microelectrode and the final endpoint position of a DBS electrode, but migration is known to occur. Finally, the correlation of high mechanical accuracy with outcomes in addition to understanding that correlation with the final endpoint of DBS electrode position is an important direction for future studies.
